# Supplementary material for: Are carbon emissions trading and green financial instruments synergistic? -Comprehensive quantitative research based on content analysis
Source: PLoS One. 2024 Mar 7;19(3):e0298601. doi: 10.1371/journal.pone.0298601 (PMC10919601; doi:10.1371/journal.pone.0298601)
Supplement: S1 Data — (ZIP) [file pone.0298601.s001.zip › Data/地级市Z_F工作报告.zip!/╦─┤¿/╫╩╤⌠╩╨/└┤╫╘╣1⁄2╓┌║┼í╛┬φ┐╦╩2╛▌═°í┐_2017.docx]

各位代表：

现在，我代表市人民政府向大会报告工作，请予审查，并请市政协委员和其他列席人员提出意见。

2016年和本届政府工作回顾

2016年，在省委、省政府和市委坚强领导下，在市人大、市政协监督支持下，我们紧紧依靠全市人民，始终保持专注发展定力，推动资阳发展取得新成绩，一批重点工作实现突破：成资一体化翻开新篇章，开创了资阳开放合作新局面；成都地铁18号线延伸到资阳、蓉昆高铁经过资阳，将迎来地铁、“双高铁”新时代；资阳临空经济区正式获批，成为引领未来发展的强大引擎；全省首台新能源空铁列车在资阳下线，共享铸造3D打印实现产业化应用，口腔装备材料产业从无到有，先进制造业发展取得突破性进展；前后科技西南电商城、各县区电子商务示范县加快建设，新兴产业培育力度加大；成安渝高速公路资阳段建成通车，万达广场开业运营，凤岭公园、沱西滨江景观带等重大项目开工建设，群众多年的期盼正变为现实；“十大专项治理行动”深入推进，市容市貌实现明显变化；华西医院、省人民医院入驻资阳，奠定了医疗水平跨越提升的坚实基础；82个贫困村退出、5.08万贫困人口脱贫，脱贫攻坚首战告捷。

一年来，我们主要做了以下工作：

——全力以赴稳增长，经济保持平稳较快发展势头。预计实现生产总值935亿元、增长8%左右，地方一般公共预算收入46.7亿元、同口径增长9%，城乡居民人均可支配收入分别增长8%、10%，经济运行呈现总体平稳、稳中有进的良好态势。全面落实政策措施，深入贯彻国、省稳增长政策，出台工业、服务业、金融、房地产等稳增长措施，全力支持实体经济发展，为企业争取专项支持1.1亿元，政策性减税9.2亿元。强力推进“项目年”活动，前后科技等66个重点项目开工建设，渝成制鞋等97个重点项目加快推进，贵均卫材等18个重点项目竣工投用，5个PPP项目启动实施，预计全社会固定资产投资完成782亿元、增长7.5%左右。大力培育产业支撑，扎实推进工业“五大攻坚”，预计规模以上工业增加值增长9.2%左右，高新技术产业产值375亿元，工业投资176亿元，经济开发区获批长江经济带国家级转型升级示范开发区；深入实施服务业“五大行动”，预计实现增加值273亿元、增长9%左右，社会消费品零售总额完成325亿元、增长10.4%左右，电子商务发展走在全省前列，渤海银行成功入驻，金融机构存贷款突破1600亿元；突出现代农业“五大抓手”，预计实现增加值154亿元、增长3.8%，粮食生产“十连增”，建成250个幸福美丽新村。持续深化改革扩大开放，行政审批、商事登记、农业农村等改革稳步推进，在全省率先实施城乡医保“三项制度”整合；完成内陆开放前沿区规划编制；预计到位国内省外资金310亿元，实现进出口1.1亿美元。

——坚定不移打基础，支撑长远发展的条件不断改善。着力提升城镇品质，完成资阳城市总规修编和总体城市设计工作；强力实施“改造完善提升攻坚年”行动，预计全市完成城市基础设施投资115亿元；安岳、乐至城市规模不断扩大、城市功能不断完善，城市管理水平不断提升，全市11个国省重点小城镇加快建设。持续改善基础条件，资潼高速公路启动建设，资三和资安快速通道前期工作有序开展，改造县乡道197公里，建成村社道864公里，预计完成交通投资80亿元；毗河供水一期工程完成投资15亿元、二期工程前期工作有序推进，预计完成水利投资30.4亿元；新扩建110千伏以上变电站3座，全面实现城乡居民同网同价，每年减少农村居民电费1亿元。加强生态环境保护，在全省率先实施大规模绿化全市行动，森林覆盖率达到39%，坚决向污染宣战，大力整治大气、水、畜禽养殖污染，耕地和基本农田保护严格控制在红线以内。

——坚持不懈补短板，改善社会民生迈出坚实步伐。深入推进脱贫攻坚，落实生产和就业、易地扶贫搬迁、兜底保障等精准扶贫措施，创新推行贫困人口全程包干救治机制。大力实施民生工程，省下九项民生工程、16件民生大事和市确定的16件民生实事全面完成，民生支出占地方一般公共预算支出的66%。协调发展社会事业，开工建设公办幼儿园14所，实施农村薄弱学校改造项目262个、化解学校大班额项目16个；深入推进公立医院改革，全面取消药品加成，市第一人民医院、精神病医院创“三甲”通过评审；市游泳综合训练馆、“非遗”展示中心建成投用。外事侨台、统计、档案、供销、民族宗教、地方志、气象、红十字、老龄、残疾人、妇女儿童等工作取得新成绩。

——多措并举防风险，攻坚化解矛盾问题取得积极进展。深入开展“走企业、解难题”活动，58户企业反映的95个问题、52个项目推进中的69个问题逐一解决；突出破解资金难题，市、县应急转贷金直接转贷10.7亿元，帮助企业贷款85亿元，发行企业债券24.5亿元。持续优化金融生态环境，依法查处逃废银行债务、非法集资等违法行为，不良贷款和不良贷款率实现“双降”。务实解决城建领域问题，全力化解毛地拍卖项目、问题楼盘等矛盾问题，“三年攻坚行动”锁定的政府性安置房全部开工建设，竣工277万平方米。积极化解政府债务，全市置换和偿还债务160.6亿元，其中市本级34.9亿元。切实维护社会和谐稳定，深入推进依法治市，扎实开展法律“七进”，深化立体社会治安防控体系建设，市本级信访下降35.7%，安全生产、食品药品安全形势稳定可控，国防动员、国家安全、应急管理、保密和人民防空工作全面加强。

——持之以恒提效能，政府自身建设切实加强。坚决服从市委领导，自觉接受人大、政协和社会各方面监督，人大代表建议、政协提案办理质量不断提高。坚持依法行政，严格按照法定权限履行职责、推动发展。全面加强政府制度建设，政府运行规范、高效。创新推进投资项目并联审批“三个集装箱”制度，审批效率进一步提高。持续改进工作作风，整治为官不为、执行不力、效率不高等问题取得阶段性成效。严格落实中央“八项规定”精神和省市“十项规定”，认真履行党风廉政建设“两个责任”，强化监察审计监督，维护了廉洁政府的良好形象。

回顾本届政府任期的五年，是经济平稳较快发展、基础条件极大改善、社会事业全面进步、人民生活水平明显提高、社会大局和谐稳定的五年。

五年来，我们始终保持专注发展定力，推动实现六个翻番，综合实力显著增强。生产总值年均增长10.3%、高于全省1个百分点，固定资产投资、服务业增加值、地方一般公共预算收入、税收收入、农民人均可支配收入、存贷款余额等六项指标成倍增长，实现了新常态下有速度、有质量的发展。先后获得全国创新社会治理优秀城市、全国双拥模范城和省级环保模范城市、园林城市等20余项荣誉，资阳在全国全省影响力进一步扩大。

五年来，我们牢牢把握主攻方向，推动实现四大突破，发展支撑更加坚实。产业发展加速突破，四川现代、加多宝等一批重大项目建成投产，造车等优势产业持续壮大，电子商务、现代物流等现代服务业加快发展，新型农业经营主体培育、现代畜牧业发展全省领先。城乡建设强力突破，城镇建成区面积新增22.1平方公里、达到94.6平方公里，城镇化率达到39.5%，九曲河综合整治基本完成，城市新区初具规模，新村建设展露新颜。交通水利跨越突破，交通投资年均超过百亿元，成渝高铁和内遂、遂资眉高速公路建成通车，实现“县县双高速”，国省干线路况指数跃居全省第四，农村公路“硬化到乡”“村村通达”；水利投资大幅攀升，毗河供水一期工程、关刀桥水库等重点项目开工建设，旱区人民半个世纪的期盼即将变为现实。招大引强持续突破，引进21户“三类500强”企业投资项目30个、金融机构15家，招商引资成果位居全省前列。

五年来，我们更加注重质量效益，推动实现四大提升，发展步入良性轨道。发展质量稳步提升，财政收入快速增长，税收占比稳步提高，民营经济发展壮大，预计三次产业结构由23.1：54.3：22.6优化为16.2：54.4：29.4。创新能力不断提升，专利申请年均增长21.2%，建成国家高新技术企业30户，国家级实验室1个、国省企业技术中心24家。品牌效应持续提升，创建中国驰名商标6个、中国名牌1个，四川著名商标12个、四川名牌46个，国家地理标志保护产品8个，安岳柠檬进入全国果品区域品牌50强。县域经济跨越提升，雁江、安岳、乐至在全省类区综合实力排名持续上升，均被评为全省县域经济发展先进县。

五年来，我们高度重视社会民生，推动实现四大进步，民生福祉持续改善。群众生活日益改善，全面完成省下民生工程、民生大事，办成一批民生实事，财政民生支出469.3亿元，是前五年的2.3倍，新增就业7.3万人，脱贫25.32万人，城乡居民收入年均分别增长10.5%、11.9%。社会事业长足进步，办学条件明显改善，教育教学质量持续提升，高等教育办学实现零突破，公共卫生、医疗服务能力明显提高，城乡公共文化服务体系不断完善，群众性体育活动蓬勃开展，养老服务体系基本建成，社会保险法定人群实现全覆盖。生态建设持续加强，全面完成总量减排任务，创建国省级生态乡镇16个，环境质量不断改善。社会治理成效明显，依法治市全面实施，平安建设不断深化，社会治安秩序保持良好，信访工作连续8年全省先进，安全生产工作连续5年全省优秀，食品药品安全形势稳定向好，社会大局和谐稳定。

各位代表，五年来，我们经受住了考验，战胜了困难挑战，为今后发展打下了坚实基础、积蓄了持久动力。成绩的取得，是省委、省政府和市委坚强领导的结果，是市人大、市政协监督支持的结果，是全市干部群众团结拼搏的结果！在此，我代表市人民政府，向各位代表、政协委员，向各民主党派、工商联、无党派人士，向全市广大干部群众、政法干警、驻资部队官兵、民兵预备役人员，向所有关心支持我市发展的老领导、各界人士，表示衷心的感谢并致以崇高的敬意!

在总结成绩的同时，我们也清醒地认识到，全市经济社会发展仍面临不少困难和问题：经济下行压力依然较大，实体经济困难较多；重大项目储备不足，投资后劲乏力；先进制造业发展不快，服务业亟需更大突破；城镇化水平不高，建设领域矛盾问题仍然突出；社会事业欠账较多，离群众期盼还有较大差距；政府作风仍需持续改进。这些困难和问题，我们将高度重视，采取有力措施，认真加以解决。

未来五年的总体要求和目标任务建议

回首过去，我们砥砺前行、硕果累累；展望未来，资阳机遇难得、正当有为。国省利好政策叠加发力，“一带一路”、长江经济带、新一轮西部大开发战略深入实施，成渝经济区加快建设，四川获批自贸试验区，为资阳发展带来广阔空间、更多机会；成资一体化激发强大活力，成资合作的重要平台和一批引领性项目陆续启动，全面全域融入成都发展的格局正在形成；综合交通建设催生巨大动能，建设天府国际机场，“7高7轨14快”立体综合交通网络正在加快构建，必将更加凸显资阳区位优势，吸引更多资源要素涌入；优势开放平台蕴含极大潜力，临空经济区、中韩创新创业园等开放平台加快建设，必将促进先进制造业和高端服务业集聚发展；更为重要的是，经过建市以来全市人民的团结奋斗，资阳发展基础更加坚实，发展后劲持续提升，干部群众和衷共济，形成了激情奋进、共促跨越的强大合力，资阳蓄势勃发、加速崛起的时机已经到来！机不可失，时不我待。我们必须抢抓机遇、铆足干劲，向着“第一个百年”梦想昂首迈进！

今后五年政府工作的总体要求是：深入学习贯彻习近平总书记系列重要讲话精神，统筹推进“五位一体”总体布局，协调推进“四个全面”战略布局，认真落实省委、省政府治蜀兴川方略和市第四次党代会部署，保持专注发展定力，大力实施开放兴市产业强市战略，坚持改革创新驱动发展，全面推进成资一体化，加快建设成渝制造业核心区、现代服务业集聚区、内陆开放前沿区，决战决胜全面小康，奋力实现资阳发展新跨越。

主要奋斗目标是：到2021年，实现“五突破四优化三下降两翻番”。

——“五突破”：生产总值突破1500亿元、人均超过全省平均水平，全社会固定资产投资突破1150亿元，第一、二、三产业增加值分别突破210亿元、790亿元、500亿元。

——“四优化”：产业结构持续优化，第三产业占比33%；投资结构持续优化，民间投资占比55%，产业投资占比27%；工业结构持续优化，先进制造业占比50%，高新技术产业占比30%；财政收入结构持续优化，税收占比70%。

——“三下降”：万元GDP综合能耗下降16%，污染物排放总量下降到省控目标以内，PM2.5浓度下降16.8%。

——“两翻番”：财政收入、居民人均可支配收入翻番。

主要任务是：围绕市第四次党代会确立的“打造新兴增长板块，构筑开放产业高地，建设美丽怡然资阳”发展蓝图，全力建设城乡一体、协调发展的现代产业城市、交通枢纽城市、生态宜居城市、开放前沿城市、平安幸福城市。

一、建设现代产业城市。大力实施产业强市战略，构建以新兴产业为先导、先进制造业为引领、现代服务业为支撑、现代农业为基础的产业体系，建设特色鲜明、优势突出、富有竞争力的现代产业城市。

再造工业发展新优势。强力推进工业转型升级，构建“一核一极三区”发展布局，推动总量突破、结构优化、效益提升、投资倍增，实现百亿企业、上市企业“零突破”，加快建设成渝制造业核心区和先进制造强市，奋力实现工业化初期向中期跨越。着力提升主导产业。壮大机车、汽车龙头企业，大力发展新能源汽车、大功率电力机车、空铁列车等交通装备制造，引进培育发动机、变速箱、电控及零部件配套企业，推动轨道交通和商用车制造迈向高端化、国际化，建设国家机车商用车制造和出口基地，创响“西部车城”品牌。着力提质优势产业。大力发展航空食品、速冻食品等加工配送，建设面向国际市场和成渝地区的绿色食品制造配送基地；大力发展生物医药产业，建设西部医药生产研发基地。着力提速新兴产业。与国际国内口腔行业协会和知名企业深度合作，打造全国有影响力的全产业链口腔装备材料产业基地；发展临空制造、电子信息、节能环保等高端成长型产业，提升天然气开发利用水平，建设清洁能源生产基地。着力实施科技创新。强化企业创新主体地位，鼓励引导企业加大研发投入，促进新技术、新工艺、新模式应用，加快科技成果产业化。

推动服务业跨越突破。加快发展现代服务业，构建“一核两区四基地”发展布局，努力建设成渝现代服务业集聚区。打造西部航空物流基地。大力发展临空服务业，突出发展航空物流、铁路物流、公路物流，争创全国物流标准化试点市、现代物流创新发展试点市。打造西部电子商务基地。培育电商市场主体，建设电商产业园，构建电商服务体系，建设国家级电商示范市、四川跨境电商示范市。打造成渝休闲度假旅游基地。优化“一心三区三带”布局，抓好国家级旅游业改革创新先行区建设，争创 2 个 5A 级、4 个 4A 级旅游景区，建成一批乡村旅游公园，打响“蜀人原乡、红色帅乡、石刻之乡”旅游品牌。打造成渝生态康养基地。发挥生态资源优势，挖掘“中国长寿之乡”品牌内涵，培育一批健康养老产业园和文体专业市场，建设成资健康走廊，打造集康养、医养、文化于一体的生态康养基地。

加速农业提质增效。大力发展高效生态农业，构建“六带七区八基地”发展布局，建设成渝重要的优质农产品供给地。推进特色基地规模化。发展100万亩柠檬、100万亩蔬菜、100万只黑山羊、30万亩蜜柑、15万亩蚕桑，新建万亩莲藕、藤椒、白乌鱼等特色产业基地30个，建设世界优质柠檬生产出口基地、成渝优质果蔬生产基地、优质黑山羊养殖基地。推进示范园区标准化。建设宝森科技等20个现代农业示范园区，建成一批产业互动融合、科技水平领先、设施装备先进的标准化园区。推进农业经营组织化。积极发展家庭农场、专业大户、农民专合社、农业企业和农业服务组织，引进一批国内外高端农产品生产加工企业，提高农业产业化经营水平。推进新型业态多元化。积极开发农业多种功能，大力发展都市农业、创意农业，打造一批农业主题公园、观光园、科普园，促进农业与旅游、教育、文化、康养等产业深度融合，提高农业综合效益。推进农产品品牌化。扩大安全、优质、生态农产品有效供给，提升“资味”农产品区域品牌影响力，打造一批特色农产品品牌，建成省级农产品质量安全监管示范市。

二、建设交通枢纽城市。构建“7高7轨14快”交通网络体系，形成“铁公机一体、多线连成渝、市域大畅通”的立体综合交通格局，建设成渝经济区最大的次级交通枢纽。

打通三大通道。打通无缝对接的“机场通道”，规划建设连接天府国际机场的轨道交通、高速公路、快速通道，构建资阳—天府国际机场的通道体系；打通快速便捷的“成都通道”，推进多线连接成都主城区、天府新区的通道建设，形成与成都一体发展的交通体系；打通高效顺畅的“重庆通道”，建设连接天府新区和两江新区的便捷通道，畅通至重庆港的直通通道，构建通江达海的铁路公路水路联运系统。

构建五大网络。构建轨道交通网，建成成都地铁18号线资阳线，开工建设天府国际机场—资阳北城际铁路、蓉昆高铁、绵遂内宜铁路、雅眉资遂铁路，实现“县县有铁路”；加密高速公路网，建成资潼高速公路，开工建设乐至—大英—三台高速公路，完成乐至—资中—井研高速公路前期工作，实现“县县三高速”；完善快速通道网，建成资三、资安、临空经济区—天府国际机场北3条快速通道，建设资简、乐金、乐简等11条快速通道，全面推进国省干道提档升级；畅通农村公路网，新改建县乡道816公里、村社道2400公里；健全公共交通网，优化城市公交线路和站点布局，构建多层次公交体系，推进城乡客运一体化，提高公共交通运行效率。

三、建设生态宜居城市。构建以中心城区为引领、县城为支撑、特色小城镇为基础的“一主两副三廊四区”城镇空间布局，全面改善人居环境，建设山水相映、配套完善、畅洁绿美、充满魅力的生态宜居城市。

推进新型城镇化。显著提升中心城区品质，推进“一城四区、四区同建”，形成生产空间集约高效、生活空间宜居适度、生态空间山青水秀的城市形态。实施“改旧扩新”，全面推进老城区棚户区、城中村改造，推动城东新区、城南新区和临空经济区扩大规模、完善功能，建设宜居宜业的产业新城、城市新区；实施“疏脉通络”，改造提升城区干道，建设空轨示范线，建成沱四桥，打通断头路，全面黑化城市道路；实施“增绿扮靓”，绿化美化沱江、九曲河、铁路城区段和城区保留山体、城市节点，新建一批公园、湿地、广场；实施“提质惠民”，建设海绵城市和地下综合管廊，完善学校医院、文化体育、农贸市场等配套设施，建成市文体中心；实施“风貌塑造”，凸显山水园林特色，加强建筑设计和景观塑造，整治沿街立面风貌；实施“文化筑魂”，将“蜀人原乡”等文化元素融入城市建设，塑造彰显历史文化底蕴的建筑风貌和特色，建成博物馆、天台山公园等项目。着力建设特色城镇和美丽新村，推动安岳县城“东拓南延”发展，组团开发“三城七片”，建设成渝中部区域性中心城市和现代山水生态城市；推动乐至县城“四区统筹、四片同建”，建设成渝经济区新兴工业城市和生态宜居园林城市；高标准建设一批工业、旅游、商贸、生态特色小镇和幸福美丽新村。深化城乡环境综合治理，持续开展“十大专项治理行动”，推动向县城、重点乡镇延伸；大力实施“违建专项治理五年行动”，全面拆除城市建成区违法建设；扎实推进非正规垃圾堆放点专项治理，建成市垃圾环保发电厂，基本实现城乡垃圾减量化、资源化、无害化。全市城镇建成区面积120平方公里，中心城区达到60平方公里以上，城镇化率达到48%，争创国家园林城市、省级森林城市。

加快城乡水网建设。构建多水源、全市域的水网体系，实现缺水资阳向水润资阳转变。建设水资源保障体系，全面建成毗河供水一期工程、关刀桥水库，启动建设毗河供水二期工程、丹山水库、宝石水库，完善重点旱区水资源保障体系；建设城乡安全用水体系，建成中心城区第二水源和应急水源工程，提高安岳、乐至县城供水保障能力，发展农村规模化集中供水，确保城乡用水安全；建设农田水利基础体系，加快农业节水工程、水库配套灌区、小型农田水利建设，破解农业生产用水瓶颈。

改善城乡生态环境。实施全域绿化，扎实开展大规模绿化全市行动，打造全省丘陵地区林业示范市、省级生态市，森林覆盖率达到41.5%。推进绿色制造，着力开展以智能制造、清洁制造为重点的技术改造，发展循环经济，推进节能减排降碳，打造绿色产业链。加强生态保护，打好“蓝天”战役，推进大气污染防治，全面改善城市空气质量；打好“碧水”战役，开展沱江、阳化河等综合整治，加快城镇污水处理设施建设，国控断面水质全面达标；打好“净土”战役，加强土壤污染分类管控和治理修复，改善土壤环境质量。

四、建设开放前沿城市。深入实施开放兴市战略，着力创新突破、开放拓展、平台提升，大力发展开放型经济，建设全域开放、内引外联、活力迸发的内陆开放前沿城市。

全方位营造开放氛围。推动思想大解放，打破传统思想观念束缚，克服封闭狭隘、因循守旧、患得患失的意识，树立开放包容、诚实守信、合作共赢的理念，勇于开拓、善于创新、争创一流，形成只争朝夕、追赶跨越的开放氛围。推动体制机制大创新，建立完善投融资、创新创业、投资促进、人才培养等体制机制，坚决破除一切影响开放发展的障碍和制约，形成资源高效聚集、投资自由便利、要素保障有力的开放优势。推动环境大改善，创立更加开明务实的政策体系、便捷高效的政务服务体系、公平公正的法制保障体系，着力创建人无我有、人有我优的开放环境。

超常规推进成资一体化。主动承接成都非省会城市功能疏解，以超常的力度和措施推进发展规划、基础设施、产业布局、公共服务、生态建设“五个一体化”，共同打造合作平台，全力推进合作项目，努力实现基础设施互联互通、产业发展一体布局、城市功能协调配套、社会事业融合发展、社会保障“一卡通”。

多层次拓展合作空间。加强与长三角、珠三角、京津冀和重庆等区域交流合作，积极承接产业转移；深化与国省部门、央企省企、金融机构的战略合作，增强合作实效；与高校和科研机构建立“政产学研用”战略联盟，加快关键技术攻关和成果转化；加强与“一带一路”沿线国家和地区的交流，广泛参与国内外经贸合作。

高标准搭建开放平台。全力打造临空经济区，积极参与四川自贸试验区建设，争创综合保税区，大力发展临空产业，形成布局合理、基础成型、初具规模的高端产业集聚地。加快建设中韩创新创业园，加强与韩国在科研开发、智能制造、生物医药等领域交流合作，建设中韩开放合作试验区。做大做强经济开发区，建成国家高新技术产业园区。

大力度发展开放经济。创新招商引资方式，广泛开展各类投资促进活动，引进落地一批“三类500强”企业和项目。争取设立海关、商检办事机构，培育壮大外贸主体，开展资阳企业丝路行、“资阳造”产品博览会等活动，提高对外贸易发展水平。

五、建设平安幸福城市。坚持用发展创造幸福、幸福引领发展，增强群众获得感、舒适感、安全感、归属感，努力建设人民群众生活殷实、环境舒适、文化丰富、安定和谐的美好家园。

实施增收富民工程，让群众生活越来越好。大力实施大众创业、万众创新，推动创业带动就业。千方百计增加就业岗位，努力实现“家家有就业、人人有事做”。扎实推进精准扶贫、精准脱贫，实现贫困村全部退出、贫困人口全部脱贫。逐步提高最低工资标准，增加低收入群体收入，缩小收入差距。

实施社保助民工程，让群众越来越安心。坚持办好民生实事，完善社会保障体系，实现由参保人员全覆盖到所有人群全覆盖；健全以低保救助、特困人员救助、医疗救助为基础，临时救助和慈善救助为补充的社会救助体系；加强城乡养老服务，让群众老有所养、老有所乐；加大农村危房改造和保障性住房建设力度，逐步改善城乡住房条件。

实施健康利民工程，让群众身体越来越健康。大力提高医疗卫生服务水平，深化公立医院改革，健全公共卫生服务体系，加强全民预防保健，创建3所“三甲”医院和一批特色专科，实现“大病不出市县、小病不出乡镇”，持续推进人口均衡发展，争创国家卫生城市、国家健康城镇。完善城乡公共体育设施，深入开展全民健身活动，提高全民身体素质。

实施文教惠民工程，让群众精神文化生活越来越丰富。实现义务教育均衡发展，普及学前三年教育和高中阶段教育，大力发展职业教育，引进优质教育资源，培育一批优质学校。提高市民道德修养、法律素养、文明素质，加快社会诚信体系建设，倡导健康文明生活方式，争创国家文明城市。完善城乡公共文化服务体系，开展多样化群众文体活动，精心打造高质量本土文化节目，丰富群众精神文化生活。

实施和谐安民工程，让群众越来越有安全感。深入推进依法治市，创新社会治理，畅通群众诉求渠道，主动回应、依法解决群众诉求；强化社会治安综合治理，健全城乡一体化的社会治安防控体系；全面提升安全生产、应急管理、防灾减灾能力，加快建设法治资阳、平安资阳、和谐资阳，建成省级法治城市、食品安全示范城市、放心舒心消费城市。

实现五年宏伟蓝图，离不开全市人民的共同奋斗。我们将充分调动各方面积极性，凝聚全市人民的智慧和力量，激发干部群众的热情与干劲，为资阳发展提供强大合力；我们将充分激发县域经济活力，在政策、项目、用地等方面给予更多支持，推动县域经济加快发展、特色发展、竞相发展，为资阳发展提供有力支撑；我们将充分实施改革创新，坚定不移推进改革创新驱动发展，认真落实支持民营经济发展的政策措施，最大限度激发市场和社会活力，为资阳发展提供强劲动力；我们将充分激活资源要素，大力向上争取、对外引进，用好人才、资金、土地、能源等资源要素，为资阳发展提供坚实保障；我们将大力改善政务环境，切实转变政府职能，提高行政效率质量，创新服务群众、服务基层、服务企业的方式方法，为资阳发展营造优质高效的政务环境。

各位代表，未来五年，是资阳蓄势突破、追赶跨越的五年，是资阳扩大开放、拓展空间的五年，是资阳决战脱贫攻坚、决胜全面小康的五年。只要我们拿出勇于担当的精神、力争上游的决心、敢闯敢拼的勇气，团结一心，开拓奋进，就一定能实现五年宏伟目标，开启向“第二个百年”梦想进军的新征程！

2017年工作建议

2017年，我们将认真贯彻落实中央、省委经济工作会议精神和市委决策部署，紧紧围绕五年目标任务，坚持稳中求进工作总基调，统筹推进稳增长、促改革、调结构、惠民生、防风险，为建成全面小康奠定坚实基础。

今年主要预期目标建议为：实现生产总值1010亿元、增长8%，全社会固定资产投资增长8%，规模工业增加值增长9.2%，社会消费品零售总额增长10%，地方一般公共预算收入同口径增长8.5%，城乡居民人均可支配收入分别增长8%、9.5%，城镇化率提高1.3个百分点，万元GDP综合能耗下降3%，污染物排放总量完成省下目标任务，PM2.5、PM10浓度分别下降3%、5%。

实现上述目标任务，我们将突出抓好六个方面重点工作：

一、突出投资拉动、产业支撑，全力稳定经济增长

深入推进“项目年”活动。对接国家中长期规划、国省支持政策和重大部署，包装储备重大项目925个、PPP项目100个，总投资超过5800亿元。扎实推进177个市级重点项目，确保亿和汽车模具等75个项目开工建设，安井食品等70个项目加快推进，成渝客专站前广场等32个项目竣工投用，完成全社会固定资产投资845亿元。积极争取政府债券、企业债券、产业发展基金等支持，到位资金100亿元以上。

加快工业转型升级。实现全部工业增加值488亿元。推动商用车和轨道交通装备制造释放产能，加快食品、医药产业盘活存量提质发展，促进口腔装备材料、电子信息产业突破发展，先进制造业占比达到35%。完成四川现代增资扩股，示范推广机车公司空铁项目，创建国家机车整车设计研制中心，积极支持中小微企业发展，推动民营企业建立现代企业制度，全市产值上亿元企业达到200户，“小巨人”和成长型企业达到20户以上。推动经济开发区创建国家级高新技术产业园区，支持雁江、安岳、乐至创建省级经济开发区，新入园企业30户以上，工业集中度超过75%。加快万贯鞋业产业园等20个投资上亿元重大项目建设，引进华光口腔等口腔装备材料产业项目20个以上，工业投资增长8%。“一企一策”精准帮扶企业，落实减税降费政策，积极争取专项基金、专项债券，持续加强银企对接、应急转贷等工作，多渠道破解企业资金难题。

大力发展现代服务业。实现服务业增加值301亿元。加快建设雁江、安岳、乐至物流园区，开工建设中铁飞豹物流港，启动全国物流标准化试点市、现代物流创新发展试点市创建。出台电子商务发展政策，深化安岳县、乐至县国家级电子商务进农村综合改革试点，持续推进省级跨境电商先行先试，加快建设前后科技西南电商城。扎实推进国家级旅游业改革创新先行区建设，加快打造花溪河、圆觉洞、闲宁村等旅游集聚区，启动资阳旅游集散中心前期工作，积极举办乡村旅游活动，实现旅游总收入160亿元。建成投用中央时代广场等城市综合体，启动建设一批农贸市场、专业市场和批发市场，打造3条特色商业街、3个重点商贸镇。积极参加和开展“惠民购物全川行” “资阳产品全国行”等活动，加快发展现代金融、科技信息、养老健康、文化体育等新兴服务业，培育消费热点。

积极发展现代农业。实现农业增加值160亿元。建设新一轮现代农业重点县、国家级畜牧业绿色发展示范县、省级新产业新业态示范县，带动产业基地提质扩面。高标准建设国道321线优质果蔬等六大产业带、国家级现代农业等七大示范区、柠檬等八大特色产业基地。开工建设温氏集团生猪产业一体化项目，启动建设柑橘新品种试验示范园，加快建设圣美园等10个种养结合循环园。大力发展旅游观光、农耕体验等新业态，打造陈毅故里农耕文化等主题公园。推动农业产业化龙头企业战略重组，新培育省级龙头企业5户、省级专合组织15个，实现60%的行政村有新型经营主体。加快创建省级农产品质量安全监管示范市，加快打造“资味”农产品区域品牌，新增1个国家地理标志产品。建成幸福美丽新村204个，创建省级“四好村”200个。抓好第三次全国农业普查工作。

二、突出城乡一体、基础先行，大力改善发展条件

攻坚推进城镇建设。完成临空经济区总体规划编制，开展中心城区12个片区控规修编，用好城市规划、城市设计成果，引领城市建设发展。实施娇子大道西延线等7个“改旧扩新”项目，完成10个棚户区、城中村35万平方米改造，启动实施临空经济区核心区基础建设；实施现代大道北延线等32个“疏脉通络”项目，打通公园路延伸段、药业路、雁南路3条断头路；实施城南大道景观绿化等7个“增绿扮靓”项目，实现凤岭公园、沱西滨江景观带开园迎宾，开展国家园林城市创建；实施茶花苑农贸市场等5个“提质惠民”项目，完善城市功能配套；实施建设北路改造等10个“风貌塑造”项目，黑化城区道路32公里，整治背街小巷50条；实施博物馆、白沙坝公园、天台山公园3个“文化筑魂”项目，彰显和传承历史文化。加强安岳、乐至县城基础设施建设，完善公共服务功能，提升宜居水平；推动中和、劳动等小城镇特色发展，启动建设龙台柠檬风情小镇。推动建筑业、房地产业健康发展，全市完成城建投资350亿元。依法处置毛地拍卖项目遗留问题，继续开展问题楼盘专项整治，加快安置房欠账攻坚扫尾，竣工186万平方米。落实“城市管理提升年”举措，突出建机制、立标准、重规范，巩固扩大“十大专项治理行动”成果，打造城市管理示范街；突出抓好建成区违建治理，拆除50%违建存量；排查整治非正规垃圾堆放点，推进城乡垃圾无害化处理；全面开展山水田林路综合治理，改善城乡环境面貌。

大力推动交通突破。力争开工建设成都地铁18号线资阳线，完成蓉昆高铁、绵遂内宜铁路前期工作。加快资潼高速公路建设，实现投资40亿元。开工建设资三、资安、卧佛路、陈毅故居旅游快速通道和资阳城东客运枢纽站，加快临空经济区—天府国际机场北、资简、乐金等5条快速通道前期工作。完成国道247线大中修及科技养护示范工程。改造提升县乡道170公里，新改建村社道500公里。全市完成交通投资90亿元。

加速建设水利工程。毗河供水一期工程、关刀桥水库分别实现投资15亿元、3亿元，完成毗河供水二期工程可研报告编制。建成城区第三期引水管道，开工建设张家岩至老鹰水库输水管道，加快四合水库引水渠、鲤鱼水库扩容前期工作。推进病险水库整治、渠系配套等工程建设。全市完成水利投资25亿元。

着力改善能源信息设施。建成500千伏川渝第三通道线路工程，实施农网升级改造5200公里，全面消除农村低电压台区。加快天然气开发利用，推进天然气民用化。建成华为天府云计算中心主体工程，新改建4G基站610座，加快建设“智慧城市”。

三、突出绿色发展、生态保护，加快建设宜居家园

实施全域绿化。启动省级森林城市创建，基本完成70座城区保留山体新造林绿化，开展国省干道、县乡通道、沱江沿岸、铁路沿线和城市园林绿化美化，实施老鹰水库等6个重要水源集雨区植树造林，创建森林小镇20个，完成营造林15万亩，森林覆盖率达到39.5%。

重拳整治污染。全面打响污染防治“三大战役”，集中整治工业废气、城区露天烧烤，严格控制城市扬尘、机动车尾气，全域禁止秸秆焚烧，确保城区空气质量优良天数280天以上；加强沱江、九曲河上游、阳化河等流域水污染防治，全面取缔网箱养鱼、肥水养鱼，完成55户畜禽养殖企业污染治理，县级以上城市集中式饮用水达标率100%，推进城区截污干管高效运行，确保第二污水处理厂配套设施建成投用，加快建设乡镇污水处理厂；加强土壤环境质量监测，严格危险废弃物管理。大力发展绿色低碳产业，鼓励企业使用清洁能源，发展循环经济，减少工业污染排放。

加强耕地保护。完成永久基本农田划定，调整完善土地利用总体规划，加快推进农村土地整理、增减挂钩、工矿废弃地复垦试点等项目，实现耕地占补平衡，严守耕地保护红线。

四、突出改革创新、开放合作，充分释放发展活力

纵深推进各项改革。深化行政审批制度改革，探索相对集中行政许可权改革，提高审批效率。建立临空经济区管理体制。加快财税体制改革，完善地方公共财政框架，完成投融资平台公司整合重组。深化金融领域改革，健全财政金融互动机制，鼓励发展债券、基金等融资方式，推动资阳、安岳、乐至农商行开业运营。深化供给侧结构性改革，着力推进农业供给侧结构性改革，提高供给质量。统筹实施科技、教育、公安、卫生、文化、供销社等改革。

全面扩大开放合作。强力推进成资全面合作，完成成资一体化发展总体规划编制，启动实施一批成资合作项目，加快临空经济区、中韩创新创业园共建步伐。广泛开展与发达地区、央企省企、高校科研单位等多领域、多层次交流合作。充分利用中外知名企业四川行、进口展、渝洽会等重大经贸平台，开展临空经济、口腔装备材料、大型企业资阳行等投资促进活动，推动华夏幸福基业等项目签约落地，到位国内省外资金320亿元以上。积极推进“万企出国门”“资阳企业丝路行”活动，实现进出口1.2亿美元。

大力实施创新驱动。争取设立省级“双创”投资引导子基金，建成省级创新创业孵化园，入园企业30户以上，实现县区创新创业孵化园全覆盖。大力发展高新技术产业，高新技术企业达到35户、产值突破400亿元。加快10个技术创新产业化项目建设，推动一批科技成果转化。

五、突出民生优先、稳定和谐，着力提高幸福指数

坚决打赢脱贫攻坚战。统筹推进贫困村基础建设、住房改造、产业发展，全面落实教育、医疗、就业、金融等扶贫措施，确保80个贫困村退出、4.7万贫困人口脱贫。

持续用力改善民生。全面完成省下民生工程和民生大事，着力办好市确定的16件民生实事，新增城镇就业1.45万人；新增养老床位3450张；改造农村危房8800户；加大社会保障和救助力度，城镇职工基本养老保险、基本医疗保险参保率分别达98%、99%以上，完善医疗救助“一站式”结算平台；开工建设市民服务中心、迁建雁江区殡仪馆，完善乡镇（社区）便民服务中心（站），整治乡镇敬老院安全隐患。

全面提升公共服务。推动义务教育均衡发展通过评估验收、教育信息化实现“三通”、大班额化解60%以上；全面改善40所义务教育学校办学条件，开工建设四川针灸学校、华西口腔职业学院等一批项目。广泛开展全民健身活动，开工建设市文体中心，建成全民健身中心，新建城区保留山体健身步道16公里。发挥华西医院、省人民医院、华西第二医院的引领作用，辐射带动全市医疗服务水平整体提升，实现远程协同诊疗延伸到县乡；启动国家卫生城市和健康城镇创建，争创1个国家级、3个省级重点专科。办好“蜀人原乡”文化艺术周活动，建成700个村级综合文化室，创建省级文明城市。建成市老干部活动中心（老年大学）。

全力维护和谐稳定。扎实推进“七五”普法，深化拓展法律服务。主动排查化解矛盾纠纷，依法维护信访秩序，攻坚化解一批信访积案难案。运用信息科技手段提高治安防控能力，严厉打击各类违法犯罪活动。持续加强安全生产和食品药品监管，提升应急管理、救灾救济、预突处突能力，加快创建省级质量强市示范市、食品安全示范城市和放心舒心消费城市。做好国防动员工作，深化双拥创建。

扎实做好审计、统计、外事侨台、档案、民族宗教、地方志、气象、红十字、保密、老龄、残疾人、妇女儿童等工作。

六、突出依法行政、改进作风，持续提升行政效能

重法治讲规范，提高公信力。坚持依法行政，运用法治思维、法治方式推动发展、解决问题，加快建设法治政府。落实重大决策公众参与、专家论证、风险评估、合法性审查和责任追究等制度，提高依法、科学、民主决策水平。深化政务公开，让权力在阳光下运行。

重效能改作风，提高执行力。强化责任担当，狠抓工作落实，确保政令畅通、执行到位。完善政府工作规则，优化政府工作流程，确保政府运转更加规范、高效。增强效率意识、精品意识，工作快节奏、高标准、严要求，定了就干、干就干好。主动服务群众、服务企业、服务项目，提高服务水平。

重规矩守纪律，提高凝聚力。坚决服从市委领导，自觉接受人大、政协及社会各界监督，提高人大代表建议、政协提案办理质量；广泛听取民主党派、工商联、无党派人士建议意见，支持工会、共青团、妇联等群团组织发挥作用，凝聚发展合力。认真落实党风廉政建设责任，加强惩防体系建设，持续开展正风肃纪，从严查处各类违纪违法行为，营造风清气正的良好环境。

各位代表！宏伟蓝图催人奋进，跨越征程任重道远。让我们在省委、省政府和市委的坚强领导下，在市人大、市政协的监督支持下，紧紧依靠全市人民，坚定信心、振奋精神，团结拼搏、锐意进取，为决胜全面小康、实现资阳发展新跨越而努力奋斗！
